# Outcomes with chimeric antigen receptor t-cell therapy in relapsed or refractory acute myeloid leukemia: a systematic review and meta-analysis

**DOI:** 10.3389/fimmu.2023.1152457

**Published:** 2023-04-24

**Authors:** Moazzam Shahzad, Andrea Nguyen, Ali Hussain, Mohammad Ammad-Ud-Din, Muhammad Salman Faisal, Ezza Tariq, Fatima Ali, Atif Butt, Iqra Anwar, Sibgha Gull Chaudhary, Forat Lutfi, Nausheen Ahmed, Anurag K. Singh, Peiman Hematti, Joseph P. McGuirk, Muhammad Umair Mushtaq

**Affiliations:** ^1^ Division of Hematologic Malignancies & Cellular Therapeutics, University of Kansas Medical Center, Kansas City, KS, United States; ^2^ Moffitt Cancer Center, University of South Florida, Tampa, FL, United States; ^3^ Division of Hematology/Oncology, Roswell Park Cancer Institute Buffalo, NY, United States; ^4^ Division of Hematology/Oncology, University of Wisconsin School of Medicine & Public Health, Madison, WL, United States

**Keywords:** acute myeloid leukemia, relapsed or refractory AML, chimeric antigen receptor T cell therapy, outcomes, immunotherapy

## Abstract

**Background:**

We conducted a systematic review and meta-analysis to evaluate outcomes following chimeric antigen receptor T cell (CAR-T) therapy in relapsed/refractory acute myeloid leukemia (RR-AML).

**Methods:**

We performed a literature search on PubMed, Cochrane Library, and Clinicaltrials.gov. After screening 677 manuscripts, 13 studies were included. Data was extracted following PRISMA guidelines. Pooled analysis was done using the meta-package by Schwarzer et al. Proportions with 95% confidence intervals (CI) were computed.

**Results:**

We analyzed 57 patients from 10 clinical trials and 3 case reports. The pooled complete and overall response rates were 49.5% (95% CI 0.18-0.81, I^2 ^=65%) and 65.2% (95% CI 0.36-0.91, I^2 ^=57%). The pooled incidence of cytokine release syndrome, immune-effector cell associated neurotoxicity syndrome, and graft-versus-host disease was estimated as 54.4% (95% CI 0.17-0.90, I^2 ^=77%), 3.9% (95% CI 0.00-0.19, I^2 ^=22%), and 1.6% (95%CI 0.00-0.21, I^2 ^=33%), respectively.

**Conclusion:**

CAR-T therapy has demonstrated modest efficacy in RR-AML. Major challenges include heterogeneous disease biology, lack of a unique targetable antigen, and immune exhaustion.

## Introduction

Acute myeloid leukemia (AML) accounts for only 1% of all new cancers in the United States and remains one of the most aggressive hematological malignancies in adults with a 5-year survival rate of 30.5% (1). The prognosis of AML is poor, with a cure rate of a mere 5-15% in patients above age 60 years, and 35-40% in patients younger than 60 years (2). At the time of diagnosis, in most cases the disease initially responds to high-dose induction chemotherapy (3); nevertheless, 10-40% of patients are primarily refractory to induction chemotherapy (2). Allogenic hematopoietic stem cell transplant (HSCT) is deemed as the only definitive treatment for AML at this time for intermediate and high-risk patients, as it is expected to result in long-lasting complete remission (CR) (4). However, 50% of patients that undergo HSCT and 80% of the patients that are not eligible or while waiting for HSCT will eventually relapse and die of the disease (5). The treatment options for the relapsed disease are limited and median overall survival is in months after the disease relapse (6).

Chimeric antigen receptor (CAR)-T cell therapy has shown promising results for the treatment of chemotherapy-refractory B cell malignancies, including acute lymphoblastic leukemia (ALL) and B cell lymphoma as well as for multiple myeloma (7–10). The use of CAR-T cell therapy is now being investigated for the treatment of AML (11). The road to successful introduction and incorporation of CAR-T cell therapy to the treatment of AML has some key roadblocks that include, but are not limited to, antigenic heterogeneity that is prevalent in AML; the AML tumor microenvironment; and on-target off-tumor toxicities (12). In this paper, we bring forth the current advances made in CAR-T cell therapy for AML treatment as found in published data in the form of case reports, case series, and clinical trials.

## Methods

### Data source and search strategy

A detailed literature search was performed for the systematic review and meta-analysis following the Preferred Reporting Items for Systematic Reviews and Meta-Analysis (PRISMA) guidelines. A population, intervention, comparison, and outcome table were developed, and three electronic databases (PubMed, Cochrane Register of Controlled Trials, and Clinical trials.gov) were searched using MeSH terms and keywords for “Leukemia, Myeloid, Acute” AND “Receptors, Chimeric Antigen” OR “adoptive immunotherapy” from the date of inception to December 6, 2022. No filters or publication time limits were applied for the search. We also searched conference abstracts of annual meetings e.g., American Society of Hematology, American Society of Clinical Oncology, and American Society of Transplantation and Cellular Therapy. A total of 677 records were identified using the database search. All search results were imported to the Endnote X9.0 reference manager, and duplicates were removed.

### Selection criteria

**Figure 1:**
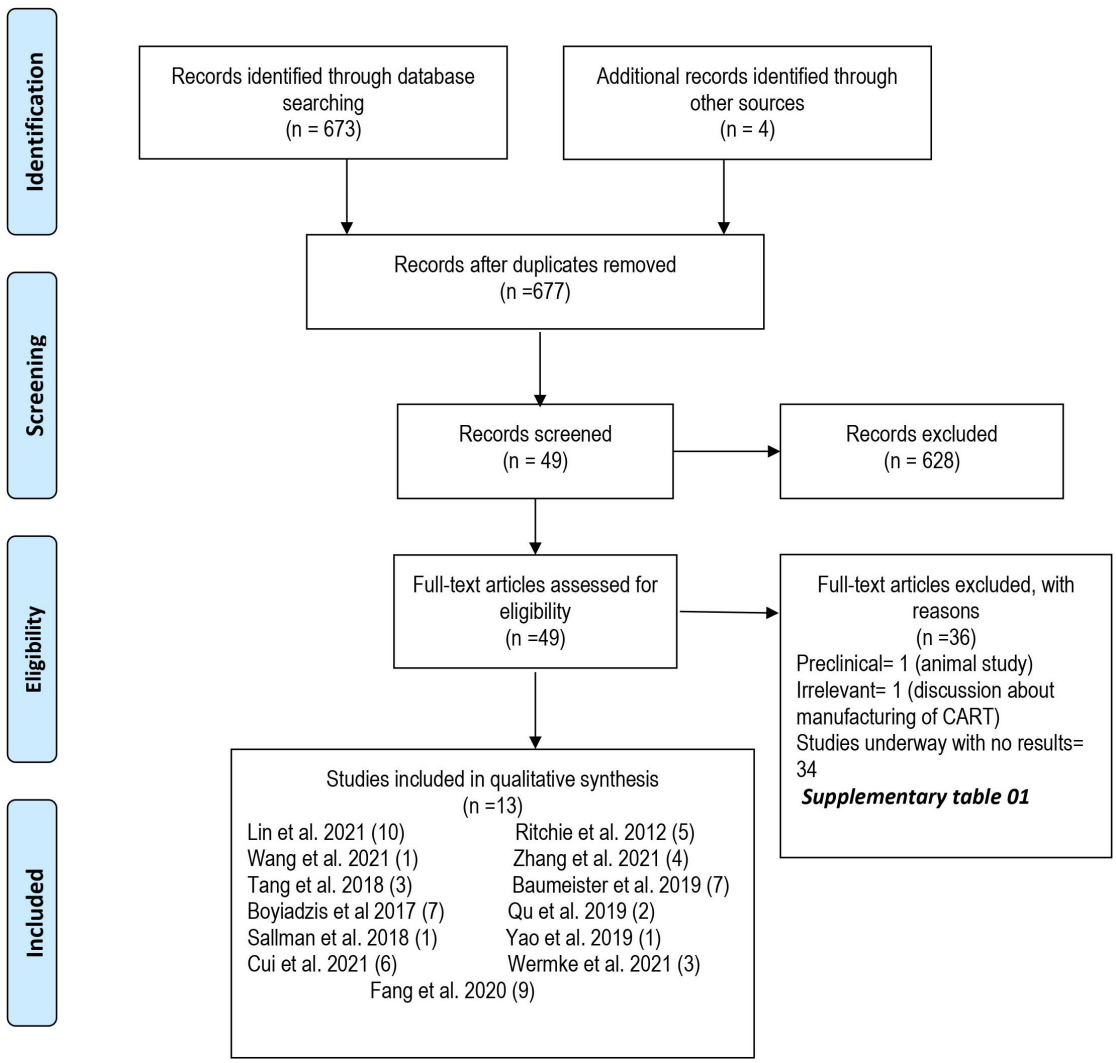
PRISMA Flow sheet of included and excluded studies.

A total of 677 articles were screened independently by two authors. In primary screening, we excluded nonrelevant and review articles. Full texts of the remaining 49 articles were then assessed for eligibility based on predetermined criteria that were set after discussion and consensus between all authors and approved by the principal investigator (M.U.M.). Inclusion criteria were (1) original studies (clinical trials and case-control, retrospective, and prospective cohort studies); (2) studies reporting data for any age; (3) studies reporting only R/R AML patients; and (4) studies reporting CAR-T therapy as the intervention. A total of 13 studies (10 clinical trials and 3 case reports) were included, and 36 studies were excluded in secondary screening based on inclusion criteria (**Figure 1**). **Supplementary Table S1** lists excluded studies along with the reasons for exclusion.

### Data extraction

Three authors independently extracted data from the 13 selected studies. Datasheets were double-checked for any discrepancies. Data were collected on baseline characteristics (i.e., number of patients, sex, age, diagnosis, prior and subsequent HSCT, and conditioning therapy before CAR-T) and the following outcomes were extracted: CR, partial response (PR), overall response rate (ORR), overall survival (OS), progression-free survival (PFS), stable disease (SD), progressive disease (PD), cytokine release syndrome (CRS), immune-effector cell associated neurotoxicity syndrome (ICANS), and graft-versus-host disease (GVHD). The response was defined as per the clinical trials or the case reports and was not uniform. The instances where response assessment was not mentioned were excluded from the analysis.

### Quality evaluation

The methodological quality of the included studies was evaluated using the National Institute of Health NIH quality assessment tool.

### Data analysis

The inter-study variance was calculated using the Der Simonian-Laird Estimator. Proportions along with a 95% confidence Interval (CI) were extracted to compute pooled analysis using the ‘meta’ package by Schwarzer et al. in the R programming language (version 4.16-2).

## Results

We identified 57 patients in 13 studies (3 case reports and 10 clinical trials) who received CAR-T therapy for RR-AML (**Table 1**). The median age of patients was 41 (7-80) years (13–25). Twelve studies mentioned sex ratio and 64% (n=32/50) were male (13, 15–25). Twenty-nine percent (12/41) of patients had a history of allogeneic HSCT before CAR-T therapy (13, 15–24), while subsequent HSCT was performed in 19% (11/57) of patients (13–25). Lin et al. did not specify the source of CAR-T cell therapy (autologous vs allogeneic) (13), while it was autologous in 33/47 (70%) and allogeneic in 14/47 (30%) of the patients (14–25). Eight studies were conducted in China (13, 16–18, 22–25), three in the US (14, 19, 20) and one study in Germany (21) and Australia (15) each. Five studies used fludarabine and cyclophosphamide (FluCy) as conditioning regimen (15, 18, 21, 23, 25) while Que et al. used decitabine with FluCy for conditioning (24). Four studies did not use any conditioning (13, 14, 17, 20), and two studies did not report regarding the conditioning regimen (16, 19). The target antigens were CD123 (21, 22), NKG2D (14, 20), CLL-1 (13, 18), CD33 (16, 17), CD33-CLL1 (25), CD19 (24), CD33, CD34, CD45, CD117 (19), CD38 (23), and Ley Ag (15).

**Table 1:** Baseline Characteristics of chimeric antigen receptor T-Cell (CAR-T) therapy in acute myeloid leukemia (n=57).

Baseline Characteristics	Lin et al. 2021	Ritchie et al. 2013	Wang et al. 2015	Zhang et al. 2021	Tang et al. 2018	Baumeister et al. 2019	Boyiadzis et al. 2017	Qu et al. 2019	Sallman et al. 2018	Yao et al. 2019	Cui et al. 2021	Wermke et al. 2021	Fang et al. 2020
**Evaluable patients, n**	10	4	1	4	3	7	6	2	1	1	6	3	9 ^**^
**Age in years, Median (Range)**	27 (8-56)	71 (64-78)	41	8.4 (7.3-9.6)	24 (14- 49)	70 (44-79)	71 (56-80)	15, 18	52	25	34.5 (7-52)	66 (54-80)	32(6-48)
**Male gender, n (%)**	7 (70)	2 (50)	1 (100)	2 (50)	1 (33)	NA	6 (100)	2 (100)	1 (100)	1 (100)	5 (83)	3 (100)	1 (11)
**Study Design**	Clinical Trial	Phase I Clinical Trial	Clinical Trial	Phase I/II Clinical Trial	Phase I Clinical Trial	Phase I Clinical Trial	Phase I Clinical Trial	Case report	Case report *	Case report	Clinical trial	Clinical Trial	Phase I Clinical Trial
**Location**	China	Australia	China	China	China	USA	USA	China	USA	China	China	Germany	China
**AML Status**	Refractory=1Relapsed=9	Refractory=3 Relapsed=1	Relapsed & Refractory	All relapsed & Refractory	All relapsed	Refractory=4Relapsed=3	All relapsed & Refractory	Refractory=1 Relapsed=1	Relapsed & Refractory	Relapsed	Relapsed & Refractory	Relapsed and Refractory	Relapsed and Refractory
**Prior HCT**	No	No	No	No	1 Patient	NA	No	1 patient	Yes	Yes	6 Patients	2 patients	NA
**Prior Therapies Median/Drugs**	NA	See footnote A	MA, HIDAC, DA and IA	Patient 1,2 &3 (MA), Patient 4 (HA)	See footnote B	1 (0-4) Footnote C	See Footnote D	“3+7” regimen IA, HIDAC	“3+7” regimen IA, CLAG-M	DA, MA+IA, DCAG, DMA & CLAG + DLI	D+HAAG, D+ECAG	Chemotherapy, Azacytidine & Venetoclax	Chemo 8 TKI 1 (CML)
**Conditioning Regimen**	None	FC	None	FC	NA	None	NA	Decitabine & FC	None	RIC regimen of TVFB	FC	FC	FC
**Post CAR-T Allo-HCT**	No	No	No	1 Patient at day 90	1 Patient at day 60	1 patient at day 120	No	No	Yes at day 97	Yes	No	No	6
**Source of CAR-T cell therapy (Autologous vs Allogeneic)**	Auto & Allo	Autologous	Autologous	Autologous	Allogenic (NK-92 Cells)	Autologous	Allogenic (aNK cell)	Auto (1), Allo (1 from sibling donor)	Autologous	Allogeneic (donor derived)	Autologous 4, Allogeneic donor 2 (donor derived)	Autologous	Autologous 8, MSD 1
**Manufacturing time in days**	14	12	13	NA	NA	9	10	Case 1 (8) Case 2 (14)	NA	8-12 days	NA	NA	NA
**Target Antigen**	CLL-1	LeY Ag	CD33	CLL-1	CD33	NKG2D	CD33, CD34, CD45, CD117	CD19	NKG2D	CD123	CD38	CD123	CLL 1-CD33
**Transduction Mechanism**	Lentiviral vector	Retroviral	Lentiviral vector	Lentiviral vector	Lentiviral	Retroviral	NA	NA	Retroviral	Retroviral	NA	NA	NA
**Costimulatory Domain**	CD-3 and CD-28	CD28 and the TCR-ζ chain	4-1BBζ-GFP	CD28-CD27-CD3z	CD-28/4-1BB	Dap10	NA	NA	CD3ζ	41BB	41BB, CD3ζ	CD-28	NA

*Part of THINK (**TH**erapeutic **I**mmunotherapy with **NK**R-2) trail, ^**^Seven were de novo AML, one was JMML transformed AML, one was CML in accelerated phrase.

A- Patient #1: IA, Patient #2: FLA, Patient #3: IA, FLA-GO, Patient #4: IA, FLA-Mylotarg, Patient #5: IA, FLA.

B- Patient #1: IA, FLA & MA, Patient #2: HAG, FLAG & multiple other chemotherapies, Patient #3: HAG, IA, AE, & MA.

C- AML patients either received non prior therapies or a combination of DA, FLAG, HIDAC, Decitabine, AZD chemotherapies.

D- Patient #1: IA, High dose Cytarabine, Dasatinib & ATRA, Patient #2: IA, FLA, ME, CFA, Patient #3: IA, ME, FLA, Patient #4: IA&CD Patient #5: CD, DAZ, Patient #6: CD, Patient #7: CD.

CLL-1, C-type lectin-like molecule 1; Ag- Antigen; NKG2D- Natural Killer Group 2D; NA, not available; FC- Fludarabine & Cyclophosphamide; TVFB- Therarubicin, Teniposide, fludarabine & busulfan, HSCT- Hematopoietic stem cell transplant, Allo- Allogenic, FLA-, Fludarabine, Cytarabine(Ara-C), CD- Cytarabine & Decitabine, DAZ- Decitabine & Azacytidine, CFA- Clofarabine & Cytarabine, FLAG- Fludarabine, Cytarabine & G-CSF, AZD- Azacytidine, GO- Gemtuzumab Ozogamicin, HIDAC- High-dose intermittent ARA-C, MA-mitoxantrone & cytarabine, ME- mitoxantrone & Etoposide, AE- Cytarabine & Etoposide, HA- Homoharringtonine & Cytarabine, HAG- Homoharringtonine, Cytarabine & G-CSF, IA- Idarubicin & Cytarabine, DA- Daunorubicin & Cytarabine, CLAG-M-cladribine, cytarabine, G-CSF & mitoxantrone, DCAG- Decitabine, Cytarabine, Aclacinomycin & G-CSF, DMA- Decitabine, Mitoxantrone & Ara-C, DLI- Donor lymphocyte infusion, D+HAAG- Decitabine, Homoharringtonine, Cytarabine, Aclarubicin, G-CSF, D+ECAG- Decitabine, Etoposide, Cytarabine, Aclarubicin, G-CSF.

**Figure 2:**
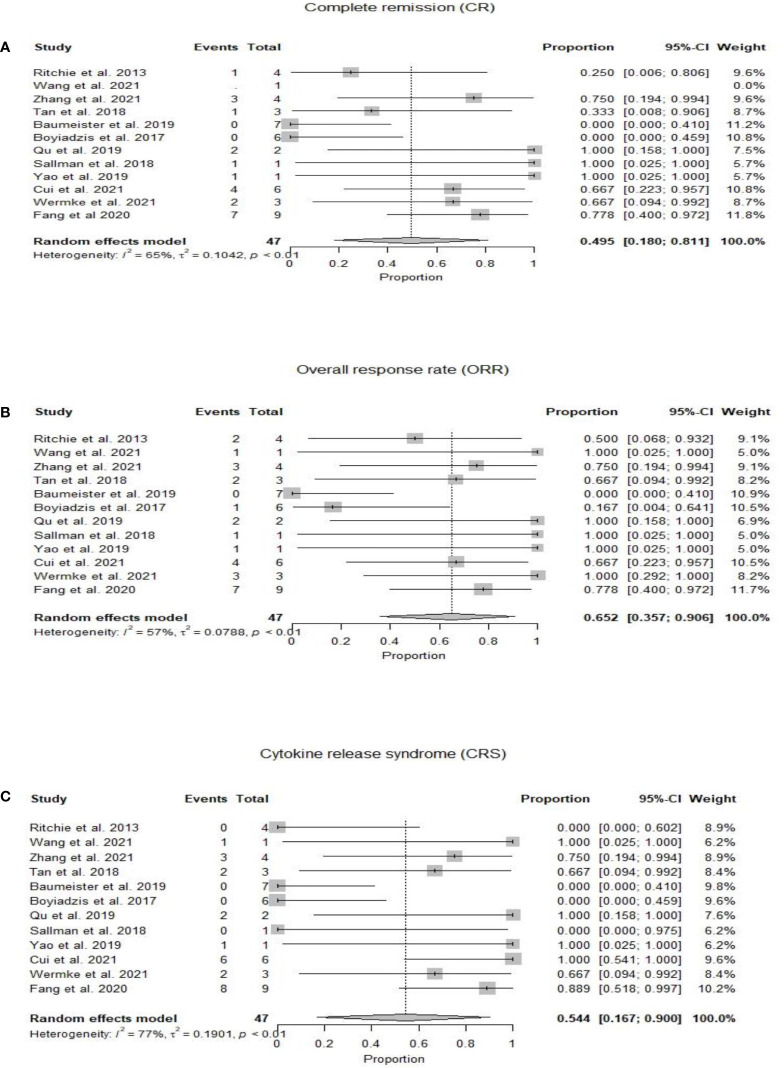
**(A)** Forest plot of complete response rate (CR) post CAR-T therapy for relapsed refractory acute myeloid leukemia. **(B)** Forest plot of overall response rate (ORR) post CAR-T therapy for relapsed refractory acute myeloid leukemia. **(C)** Forest plot of cytokine release syndrome (CRS) post CAR-T therapy for relapsed refractory acute myeloid leukemia.

At a median duration of 5.7 (1-23) months (15–25), OS was reported from 1.8 months (22) to 23 months (18). Mean OS could not be calculated due to heterogeneity of the included studies (**Table 2**). Twenty-two patients had complete remission after CAR-T therapy, with an estimated pooled incidence of 49.5% (95% CI 0.18-0.81, I^2 ^=65%) (14–25) (**Figure 2A**). The pooled incidence of ORR was estimated as 65.2% (95% CI 0.36-0.91, I^2 ^=57%) (14–25) (**Figure 2B**). One out of the four included patients in Ritchie et al. remained without cytogenetic disease for 23 months, while the other three patients relapsed on day 49, day 28, and at 5 months, respectively (15). Wang et al. reported OS of 3.1 months (17) while in Zhang et al. patients’ number 1, 2, and 3 died at 23, 5, and 12 months respectively, and patient number 4 was alive at 9 months. One out of the three included patients in Tang et al. relapsed at 4 months and no treatment was pursued, the second patient relapsed 6 months post CAR-T and 4 months post-HSCT and died of grade IV GVHD after salvage chemotherapy and donor lymphocyte infusions, and the third patient did not respond to CAR-T (16). At 21 days post-CAR-T, no patient achieved CR, and only one patient had a reduction in blast percentage in Boyiadzis et al. (19) Both included patients by Qu et al. achieved CR and one of them maintained CR for over 10 months, while the other refused further treatment after CR and relapsed at 3 months (24). The patient reported by Sallman et al. maintained CR for 6 months post-HSCT and 9 months following initial CAR-T cell therapy (20), while the patient in Yao et al. received CAR-T as part of conditioning therapy and achieved CR with incomplete count recovery, developed grade IV GVHD on day 32 and died on day 56 (22). Cui et al. reported the 6-month OS and leukemia-free survival (LFS) rates were both 50%, and the median OS and LFS were 7.9 and 6.4 months, respectively (23). Two patients in Wermke et al. achieved complete remission with incomplete hematologic recovery, one of which remained in remission at 100 days, while the other relapsed at one month after the CAR-T cell therapy (21). Fang et al. reported that seven out of nine patients were minimal residual disease negative (MRD^-^) at four weeks follow-up post CAR-T cell therapy. Six out of these seven MRD^-^ patients moved to subsequent HSCT with successful engraftment and persistent full chimerism in five patients. One patient died of sepsis on day +6 before engraftment (25).

**Table 2:** Outcomes with Chimeric Antigen Receptor T-Cell (CAR-T) Therapy in Acute Myeloid Leukemia (n=57).

Outcomes	Lin et al. 2021	Ritchie et al. 2013	Wang et al. 2021	Zhang et al. 2021	Tang et al. 2018	Baumeister et al. 2019	Boyiadzis et al. 2017	Qu et al. 2019	Sallman et al. 2018	Yao et al. 2019	Cui et al. 2021	Wermke et al. 2021	Fang et al 2020
**Overall response rate, n (%)**	NA	2 (50)	1 (100)	3 (75)	2 (67)	0	1 (17)	2 (100)	1 (100)	1 (100)	4 (67)	3 (100)	7 (78)
**Complete remission, n (%)**	NA	1 (25)	0	3 (75)	1 (33)	0	0	2 (100)	1(100)	1 (100)	4 (67)	2 (67)	7(78)
**Partial response, n (%)**	NA	1 (25)	1 (100)	0	1 (33)	0	1 (17)	0	0	0	1 (17)	1 (33)	0
**Follow-up, response duration and overall survival**	NA	*See footnote A*	Survival 3.1 months	*See footnote B*	*See footnote C*	NA	*See footnote D*	*See footnote E*	*See footnote F*	*See footnote G*	*See footnote H*	*See footnote I*	*See footnote j*
**Cytokine release syndrome, n (%, Grade)**	NA	0	1 (100, IV)	3 (75, I-II)	2 (66, I-II)	0	0 *	2 (100, I-IV)	0	1 (100, III-IV)	5 (83 I-II), 1 (17, III)	2 (67, I)	8(89) 3 I, 3II, 2 III
**Neurotoxicity, n (%)**	NA	0	NA	1 (25)	NA	0	0	NA	0	NA	0	0	4 (44)
**GVHD, n (%)**	NA	0	NA	NA	1 (33)	0	0	NA	NA	1 (100)	0	NA	NA

*Grade II fever and Chills.

A: Patient #1 relapsed at day 49 after CAR-T, #2 remained with cytogenetic disease out to 23 months, #3 progressed at day 28 after CAR-T, and #5 relapsed at 5 months.

B: Patient #1, 2 and 3 died at 23, 5 and 12 months respectively, #4 alive at 9 months.

C: Patient #1 relapsed at 4 months and no treatment pursued, #2 relapsed 6 months post CAR-T and 4 months post allo-HCT and died of grade IV GVHD after salvage chemotherapy and donor lymphocyte infusions, # 3 no response to CAR-T.

D: At 21 days post CAR-T, no patient achieved complete remission, 1 patient had reduction in blast percentage.

E: Patient #1 maintained complete remission for over 10 months, #2 refused further treatment after complete remission and relapsed at 3 months.

F: Patient maintained complete remission 6 months from allo-HCT and 9 months following initial CAR-T cell therapy.

G: Patient received CAR-T as part of conditioning therapy and achieved complete remission with incomplete count recovery, developed grade IV GVHD on day 32 and died on day 56.

H: Patient #4 experienced relapse 117 days after the first CAR-T-38 infusion but achieved remission after a second CAR-T-38 treatment. The 6-month OS and LFS rates were both 50%, and the median OS and LFS were 7.9 and 6.4 months, respectively.

I: Patient # 1 in remission at 100 days, Patient # 2 showed leukemia regrowth one-month post-CAR-T.

J: Seven out of nine patients were minimal residual disease negative (MRD^-^) at four weeks follow-up post CAR-T cell therapy and six out of these seven MRD^-^ patients moved to subsequent HSCT with successful engraftment and persistent full chimerism in five patients. One patients died of sepsis on day +6 before engraftment.

NA, Not available; GVHD, Graft-versus-host disease; Allo, Allogenic.

In 10 patients from 3 studies, CD33 target antigen was used (16, 17, 19). Boyiazis et al. used CD34, CD45, and CD117 in addition to CD33. Four out of 10 patients showed ORR (40%) and one patient had CR (10%). In two studies, four patients were treated with CAR-T targeting CD123 antigen (21, 22). These four patients showed ORR (100%) while three of them achieved CR (75%). NKG2D target antigen was used in eight patients from two studies, and only one out of eight patients showed CR (12.5%) (14, 20). CLL-1 was used in two studies, however, only four patients reported outcomes and three of these patients had CR (75%) (13, 18). CD33^+^-CLL1^+^ was the target antigen in Fang et al. and seven out of nine patients achieved MRD^-^ while two patients did not respond (25).

Twenty-five patients developed CRS with a pooled incidence of 54.4% (95% CI 0.17-0.90, I^2 ^=77%) (14–25) (**Figure 2C**). Only five patients in eight studies developed ICANS, with a pooled incidence of 3.9% (95% CI 0.00-0.19, I^2 ^=22%) (14, 15, 18–21, 23, 25). GVHD was reported by six studies and developed in two patients who had prior and subsequent HSCT after CAR-T therapy; one of them developed grade IV GVHD and died, in the setting of salvage therapy with donor lymphocyte infusions for relapsed disease 6 months post-CAR-T and 4 months post second HSCT (16), while the other patient received CAR-T as part of conditioning therapy and developed grade IV GVHD on day 32 post-HSCT (22). CAR-T cell products were allogeneic in both cases who had GVHD (16, 22). Qu et al. and Fang et al. reported grade IV cytopenias in all enrolled patients (24, 25) while Ritchie et al. reported grade II neutropenia in only 25% of the patients (15). Zhang et al. observed grade III anemia and grade IV neutropenia in four and three patients, respectively (18). Fang et al. reported non-hematologic toxicity as liver function test elevation (56%), coagulation disorder (44%), diarrhea (44%), skin rash (11%), and renal insufficiency (11%) (25). Fang et al. also reported incidence of pneumonia (33%), sepsis (33%), and fungal infection (22%) (25) while only one out of six patients had infection in Cui et al (23).

## Discussion

In this meta-analysis, we focused on outcomes of relapsed or refractory AML patients who underwent experimental chimeric antigen receptor T cell therapies. CAR-T cell therapy is a recent breakthrough that has shown promising results in hematological malignancies including acute lymphoblastic leukemia (7). Presently, there are 14 ongoing clinical trials in the United States (**Table 3**) and 20 active international clinical trials studying various target antigens for relapsed/refractory AML (6, 26) (**Table 4**). Examples of target antigens being investigated include CD33, CD38, CD123, UCART123, CD123/CLL1, CD33/CLL1, WT1, CD7/NK92, and NKG2D (26). Our meta-analysis included 13 studies with a total of 57 patients. Most patients were males with a median age of 41 years, and a total of 38 patients had reported outcomes. The pooled overall and complete response rate was 65.2% and 49.5%, respectively. The pooled incidence of CRS, ICANS, and GVHD was 54.4%, 3.9%, and 1.6%, respectively.

**Table 3:** Ongoing CAR-T therapy clinical trials in the United States for the treatment of relapsed or refractory acute myeloid leukemia.

National Clinical Trial (NCT) Number	Study Title	Intervention/Treatment	Phase
NCT04219163	Chimeric Antigen Receptor T-cells for The Treatment of AML Expressing CLL-1 Antigen	Biological: CLL-1.CAR T cells	Phase 1
NCT03904069	Study Evaluating the Safety, Tolerability, and Efficacy of FLT3 CAR-T AMG 553 in FLT3-positive Relapsed/Refractory AML	Drug: AMG 553	Phase 1
NCT02159495	Genetically Modified T-cell Immunotherapy in Treating Patients With Relapsed/Refractory Acute Myeloid Leukemia and Persistent/Recurrent Blastic Plasmacytoid Dendritic Cell Neoplasm	Biological: Allogeneic CD123CAR-CD28-CD3zeta-EGFRt-expressing T-lymphocytes	Phase 1
NCT03971799	Study of Anti-CD33 Chimeric Antigen Receptor-Expressing T Cells (CD33CART) in Children and Young Adults With Relapsed/Refractory Acute Myeloid Leukemia	Biological: CD33CART	Phase 1 Phase 2
NCT03766126	Lentivirally Redirected CD123 Autologous T Cells in AML	Biological: CART123 cells	Phase 1
NCT04678336	CD123 Redirected T Cells for AML in Pediatric Subjects	Biological: CART123 cells	Phase 1
NCT03927261	PRGN-3006 Adoptive Cellular Therapy for Relapsed or Refractory AML or Higher Risk MDS	Drug: PRGN-3006 T Cells	Phase 1
NCT03190278	Study Evaluating Safety and Efficacy of UCART123 in Patients With Relapsed/ Refractory Acute Myeloid Leukemia (AMELI-01)	Biological: UCART123v1.2	Phase 1
NCT04789408	Study Evaluating the Safety of KITE-222 in Participants With Relapsed/Refractory Acute Myeloid Leukemia	Biological: KITE-222	Phase 1
NCT04167696	Study in Relapsed/Refractory Acute Myeloid Leukemia or Myelodysplastic Syndrome Patients to Determine the Recommended Dose of CYAD-02 (CYCLE-1)	Biological: CYAD-02	Phase 1
NCT05377827	Dose-Escalation and Dose-Expansion Study to Evaluate the Safety and Tolerability of Anti-CD7 Allogeneic CAR T-Cells (WU-CART-007) in Patients With CD7+ Hematologic Malignancies	Biological: WU-CART-007	Phase 1
NCT05672147	CD33-CAR T Cell Therapy for the Treatment of Recurrent or Refractory Acute Myeloid Leukemia	Biological: Anti-CD33 CAR T-cells	Phase 1
NCT05442580	CART-38 in Adult AML and MM Patients	Drug: CART-38 cells	Phase 1
NCT05457010	Phase I Study of Cell Therapies for the Treatment of Patients With Relapsed or Refractory AML or High-risk MDS	Biological: SPRX002 Biological: ARC-T Cells	Phase 1

**Table 4:** Ongoing international CAR-T therapy clinical trials for the treatment of relapsed or refractory acute myeloid leukemia.

National Clinical Trial (NCT) No.	Study Title	Intervention/Treatment	Phase	Location
NCT04835519	Phase I/II Study of Enhanced CD33 CAR T Cells in Subjects With Relapsed or Refractory Acute Myeloid Leukemia	Biological: CD33 CAR-T	Phase 1 Phase 2	Beijing, Beijing, China
NCT05023707	Anti-FLT3 CAR T-cell Therapy in FLT3 Positive Relapsed/Refractory Acute Myeloid Leukemia	Biological: anti-FLT3 CAR-T	Phase 1 Phase 2	Suzhou, Jiangsu, China
NCT04923919	Clinical Study of Chimeric Antigen Receptor T Lymphocytes (CAR-T) in the Treatment of Myeloid Leukemia	Drug: Anti-CLL1 CART cells	Phase 1	Kunming, Yunnan, China
NCT04692948	TAA6 Cell Injection In The Treatment of Patients With Relapsed / Refractory Acute Myeloid Leukemia	Other: TAA6 cell injection (T cell targeting CD276 CAR-T)	N/A	Hefei, Anhui, China
NCT04257175	CAR-T CD19 for Acute Myelogenous Leukemia With t 8:21 and CD19 Expression	Biological: CAR-T CD19	Phase 2 Phase 3	Ramat Gan, Israel
NCT05432401	TAA05 Injection in the Treatment of Adult Patients With FLT3-positive Relapsed/Refractory Acute Myeloid Leukemia	Biological: T cell injection targeting FLT3 CAR	Phase 1	Wuhan, Hubei, China
NCT04884984	Anti-CLL1 CAR T-cell Therapy in CLL1 Positive Relapsed/Refractory Acute Myeloid Leukemia (AML)	Biological: anti-CLL1 CART	Phase 1 Phase 2	Suzhou, Jiangsu, China
NCT05017883	TAA05 Cell Injection in the Treatment of Recurrent / Refractory Acute Myeloid Leukemia	Drug: TAA05 cell injection targeting FLT3 CAR	N/A	Hefei, Anhui, China
NCT04762485	Humanized CD7 CAR T-cell Therapy for r/r CD7+ Acute Leukemia	Biological: Humanized CD7 CAR-T cells	Phase 1 Phase 2	Suzhou, China
NCT05266950	Safety and Efficacy Study of CI-135 CAR-T Cells in Subjects With Relapsed or Refractory Acute Myeloid Leukemia	Biological: CI-135 CAR-T cells	Phase 1	Beijing, Beijing, China
NCT04803929	Clinical Study of Anti-ILT3 CAR-T Therapy for R/R AML(M4/M5)	Biological: anti-ILT3 CAR-T	Phase 1	Hangzhou, Zhejiang, China
NCT05463640	Evaluate the Safety and Efficacy of ADGRE2 CAR-T in Patients With R/R AML	Biological: ADGRE2 CAR-T	Phase 1	Hangzhou, China
NCT05467254	Evaluate the Safety and Efficacy of CLL1+CD33 CAR-T in Patients With R/R AML	Biological: CLL1+CD33 CAR-T	Phase 1	Hangzhou, China
NCT04351022	CD38-targeted Chimeric Antigen Receptor T Cell (CART) in Relapsed or Refractory Acute Myeloid Leukemia	Biological: CART-38	Phase 1 Phase 2	Suzhou, Jiangsu, China
NCT03896854	CART-19 T Cell in CD19 Positive Relapsed or Refractory Acute Myeloid Leukemia (AML)	Biological: CART-19	Phase 1 Phase 2	Suzhou, Jiangsu, China
NCT04169022	AML Cell Immunotherapy Using Chimeric Antigen Receptor T-cells (CAR-LAM)	Other: IL1RAP CAR-T	N/A	Besançon, France
NCT05722171	Clinical Study of UTAA06 Injection in the Treatment of Relapsed/Refractory Acute Myeloid Leukemia	Biological: gdT cell injection targeting B7-H3 CAR	Phase 1	Hefei, Anhui, China
NCT04230265	Dose-escalating Trial With UniCAR02-T Cells and CD123 Target Module (TM123) in Patients With Hematologic and Lymphatic Malignancies	Drug: UniCAR02-T-CD123	Phase 1	Germany
NCT05731219	UTAA06 Injection in the Treatment of Relapsed/Refractory Acute Myeloid Leukemia	Biological: B7-H3 target, CAR gene modified gdT cell injection	Phase 1	Hangzhou, Zhejiang, China
NCT05574608	Allogenic CD123-CAR-NK Cells in the Treatment of Refractory/Relapsed Acute Myeloid Leukemia	Biological: CD123-CAR-NK cells	Phase 1	Beijing, Beijing, China

The most prominent feature of CAR-T in AML is the heterogeneity of targets used for AML. Four of the studies included in this meta-analysis had patients who underwent HSCT before CAR-T cell therapy. CD33 antigen was used in 10 of the included patients (16, 17, 19), while Boyiazis et al. used CD34, CD45, and CD117 in addition to CD33 (19). The reported ORR and CR were 40% and 10%, respectively. CD33 is a myeloid differentiation antigen that is present on myeloid blasts and has been used for antibody-based targeted therapies like gemtuzumab-ozogomycin for many years (27), so naturally, it is a common target for CAR-T development. Two studies used CD123 antigen and the reported ORR and CR were 100% and 75%, respectively (21, 22). CD123 is an alpha subunit of interleukin 3 receptor (IL3R) and is highly expressed in the AML blast cells differentially. It is expressed at a low level in hematopoietic stem cells, making it an ideal target for pharmacotherapy and cellular therapies (28). NKG2D (natural-killer group 2, member D) target antigen was used in eight patients (14, 20). NKG2D is an activating receptor that is mostly expressed on cells of the cytotoxic arm of the immune system, and NKG2D ligands are differentially expressed on malignant or stressed cells as compared to healthy tissue, making it a promising CAR candidate (29, 30). Only one out of eight patients showed CR (12.5%). CLL-1 (C-type lectin-like molecule-1) was used in two studies (13, 18); however, only four patients reported outcomes and three of these patients had CR (75%). CD33^+^-CLL1^+^ was used as target antigen in one study with 78% CR (25). Although the patient population is limited, a good response rate appears to have been observed with CD33, CLL-1, and CD-123 as target antigens. Most of the included studies did not report the type of relapse. Ritchie et al. demonstrated that LeY T cells were detectable in all patients by PCR until relapse, which ranged from 28 days to 23 months. Despite demonstrable CAR–T-cell persistence, the AML progression could be due to lost LeY expression on residual AML blasts (15).

The cumulative incidence of GVHD in CAR-T trials patient population is 5%. Twelve patients had allogeneic HSCT before CAR-T cell therapy (31.5%). Two patients in our meta-analysis who developed GVHD post-CAR-T cell therapy already had HSCT before CAR-T therapy and CAR-T cell products were allogeneic in both patients (16, 22). The contribution of CAR-T in the development of GVHD is not clear given the previous history of HSCT, and previous studies in B-ALL patients showed that GVHD is mild after allogeneic CAR-T administration (31). In the study by Liu et al., the cumulative incidence of acute GVHD after allogeneic HSCT was 39.5% (32). Another study by Sandhu et al. shows an incidence of GVHD of 43.8% (33); however, further studies are needed to better understand this.

The two major known toxicities of CAR-T cells, cytokine release syndrome and immune effector cell associated neurotoxicity syndrome (ICANS) were observed, but grade III-IV toxicities were rarely reported; only five patients had grade III-IV CRS, and none had grade III-IV ICANS. No patient died due to treatment-related toxicities. The durability of response remains a concern after CAR-T therapy and long-term follow-up data are lacking. The median duration of response for patients after CAR-T was in the range of a few months. However, Sallman et al. showed the most promising results with a patient in complete remission 6 months post-HSCT and 9 months post-CAR-T cell therapy (20). Of note, Sallman et al. also demonstrated that NKG2DL CAR-T cell therapy is not associated with significant adverse effects due to the selective up-regulation of NKG2DL on transformed cells, skipping normal hematopoietic stem cells. Ritchie et al. established that CAR-T cell persistence in blood and marrow until the time of relapse is an indication of CAR-T cell immunotherapy as a long-lasting treatment option (15).

Currently, several factors that limit the use of CAR-T therapy for AML patients, including biological barriers, manufacturing issues, limitations in the delivery of therapy, and patient-related factors (34). Most important is the lack of a target antigen that is specific to leukemic that avoids hematopoietic stem cell depletion (34). Relapsed or refractory AML patients are usually of advanced age, with limited tolerability to intensive treatment and co-morbidities restricting CAR-T therapy (34). Hostile and tolerogenic tumor microenvironment in AML can also limit the anti-tumor effectiveness of CAR-T cells resulting in immune exhaustion and relapse. Designing CAR-T cells that target antigens shared by AML blast cells and suppressive immune cells such as B7-H3 can lead to enhanced anti-leukemic activity (35, 36). Incorporating stimulatory domains such as CD28, 4-1BB, CD3, CD27, and CD3z can lead to enhanced efficacy and persistence (37, 38).

To our knowledge, this is the first meta-analysis on the safety and efficacy of CAR-T therapy for relapsed refractory AML. Study limitations include small sample size and the heterogeneous nature of the included studies. Several factors make it challenging to conduct this review including biologic heterogeneity of AML with prognostic significance, paucity of the available literature, conditioning regimens/dosing differences, and autologous versus allogeneic CAR-T constructs with a variety of targets. Additionally, due to a small number of patients and included studies, we were not able to investigate factors affecting the outcomes, such as CAR-T targets, age, prognostic markers like genetic mutations, lines of prior therapy, and prior allogeneic hematopoietic cell transplantation. Nevertheless, this seminal review will be useful to design and prioritize future clinical trials for CAR-T cell therapy in AML patients.

## Conclusion

Chimeric antigen receptor T cell therapy has shown favorable responses in relapsed/refractory acute myeloid leukemia patients with a complete remission rate observed in over one-third of patients with acceptable toxicity profile. The conclusions reached by this meta-analysis should be taken with caution given the small sample size and heterogeneous nature of the included studies. Improved CAR-T constructs will hopefully overcome current challenges, including the heterogeneous biology of AML, lack of a unique targetable antigen expression on malignant cells, and immune exhaustion, and improve the outcomes in this therapeutically challenging patient population. Therefore, further prospective clinical trials are needed to evaluate the utility of CAR-T cell therapy in this therapeutically challenging patient population.

## Author contributions

All authors contributed to the manuscript and fulfilled criteria per the uniform requirements set forth by the International Committee of Medical Journal Editors (ICJME) guidelines. All authors have reviewed and approved the final version of the manuscript. All authors contributed to the article and approved the submitted version.
