# Supplementary material for: Outcomes with chimeric antigen receptor t-cell therapy in relapsed or refractory acute myeloid leukemia: a systematic review and meta-analysis
Source: Front Immunol. 2023 Apr 24;14:1152457. doi: 10.3389/fimmu.2023.1152457 (PMC10164930; doi:10.3389/fimmu.2023.1152457)
Supplement: Supplementary file 1 [file Table_1.docx]

Supplementary Material

**Supplementary Table 1: Excluded studies in second screening with reason**

| **No** | **Authors** | **DOI/Link** | **Reason for exclusion** |
| --- | --- | --- | --- |
|  | Chet et al. 2017 | 10.1038/leu.2017.147 | Preclinical study |
|  | Murad et al. 2018 | 10.1016/j.jcyt.2018.05.001 | Study about CAR-T manufacturing |
|  | <https://ClinicalTrials.gov/show/NCT03904069> 2022 | | Clinical trial with no results |
|  | <https://ClinicalTrials.gov/show/NCT04169022> 2019 | | Clinical trial with no results |
|  | <https://ClinicalTrials.gov/show/NCT03971799> 2020 | | Clinical trial with no results |
|  | <https://ClinicalTrials.gov/show/NCT02159495> 2015 | | Clinical trial with no results |
|  | <https://ClinicalTrials.gov/show/NCT03126864> 2017 | | Clinical trial with no results |
|  | <https://ClinicalTrials.gov/show/NCT04257175> 2020 | | Clinical trial with no results |
|  | <https://ClinicalTrials.gov/show/NCT04265963> 2019 | | Clinical trial with no results |
|  | <https://ClinicalTrials.gov/show/NCT04272125> 2019 | | Clinical trial with no results |
|  | <https://ClinicalTrials.gov/show/NCT03796390> 2018 | | Clinical trial with no results |
|  | <https://ClinicalTrials.gov/show/NCT04835519> 2021 | | Clinical trial with no results |
|  | <https://ClinicalTrials.gov/show/NCT01864902> 2013 | | Clinical trial with no results |
|  | <https://ClinicalTrials.gov/show/NCT04318678> 2020 | | Clinical trial with no results |
|  | <https://ClinicalTrials.gov/show/NCT03473457> 2018 | | Clinical trial with no results |
|  | <https://ClinicalTrials.gov/show/NCT04010877> 2019 | | Clinical trial with no results |
|  | <https://ClinicalTrials.gov/show/NCT04033302> 2019 | | Clinical trial with no results |
|  | <https://ClinicalTrials.gov/show/NCT03222674> 2017 | | Clinical trial with no results |
|  | <https://ClinicalTrials.gov/show/NCT04803929> 2021 | | Clinical trial with no results |
|  | <https://ClinicalTrials.gov/show/NCT04219163> 2020 | | Clinical trial with no results |
|  | <https://ClinicalTrials.gov/show/NCT03766126> 2018 | | Clinical trial with no results |
|  | <https://ClinicalTrials.gov/show/NCT04678336> 2021 | | Clinical trial with no results |
|  | <https://ClinicalTrials.gov/show/NCT04692948> 2019 | | Clinical trial with no results |
|  | <https://ClinicalTrials.gov/show/NCT04097301> 2019 | | Clinical trial with no results |
|  | <https://ClinicalTrials.gov/show/NCT04766840> 2021 | | Clinical trial with no results |
|  | <https://ClinicalTrials.gov/show/NCT03114670> 2017 | | Clinical trial with no results |
|  | <https://ClinicalTrials.gov/show/NCT02799680> 2015 | | Clinical trial with no results |
|  | <https://ClinicalTrials.gov/show/NCT03896854> 2017 | | Clinical trial with no results |
|  | <https://ClinicalTrials.gov/show/NCT03631576> 2018 | | Clinical trial with no results |
|  | <https://ClinicalTrials.gov/show/NCT03672851> 2019 | | Clinical trial with no results |
|  | <https://ClinicalTrials.gov/show/NCT04351022> 2017 | | Clinical trial with no results |
|  | <https://ClinicalTrials.gov/show/NCT04762485> 2021 | | Clinical trial with no results |
|  | <https://ClinicalTrials.gov/show/NCT04599543> 2020 | | Clinical trial with no results |
|  | <https://ClinicalTrials.gov/show/NCT04658004> 2021 | | Clinical trial with no results |
|  | <https://ClinicalTrials.gov/show/NCT04662294> 2021 | | Clinical trial with no results |
|  | <https://ClinicalTrials.gov/show/NCT04014881> 2019 | | Clinical trial with no results |
